# The highly rearranged karyotype of the hangingfly *Bittacus sinicus* (Mecoptera, Bittacidae): the lowest chromosome number in the order

**DOI:** 10.3897/CompCytogen.v14i3.53533

**Published:** 2020-07-30

**Authors:** Ying Miao, Bao-Zhen Hua

**Affiliations:** 1 College of Life Sciences, Northwest A&F University, Yangling, Shaanxi 712100, China Northwest A&F University Yangling China; 2 College of Plant Protection, Northwest A&F University, Yangling, Shaanxi 712100, China Northwest A&F University Yangling China

**Keywords:** C-banding technique, chromosome rearrangement, cytogenetics, DAPI, evolution, Holometabola, meiosis

## Abstract

Cytogenetic features of the hangingfly *Bittacus sinicus* Issiki, 1931 were investigated for the first time using C-banding and DAPI (4',6-diamidino-2-phenylindole) staining. The karyotype analyses show that the male *B. sinicus* possesses the lowest chromosome number (2*n* = 15) ever observed in Mecoptera, and an almost symmetric karyotype with *M_CA_* (Mean Centromeric Asymmetry) of 12.55 and *CV_CL_* (Coefficient of Variation of Chromosome Length) of 19.78. The chromosomes are either metacentric or submetacentric with their sizes decreasing gradually. Both the C-banding and DAPI^+^ patterns detect intermediate heterochromatin on the pachytene bivalents of *B. sinicus*, definitely different from the heterochromatic segment at one bivalent terminal of other bittacids studied previously. The male meiosis of *B. sinicus* is chiasmate with two chiasmata in metacentric bivalents and one in the submetacentric bivalent. The sex determination mechanism is X0(♂), which is likely plesiomorphic in Bittacidae. Two alternative scenarios of karyotype origin and evolution in *Bittacus* Latreille, 1805 are discussed.

## Introduction

Bittacidae is the second largest family of Mecoptera, and currently consists of over 200 species in 18 genera in the world (Zhang et al. 2020). The adults of Bittacidae comprise an exclusive group that possesses three pairs of elongated raptorial legs with a single claw at pretarsus and adopts a predacious feeding strategy (Bornemissza 1966; Byers and Thornhill 1983; Penny 2006; Tan and Hua 2008; Ma et al. 2014). They are commonly known as hangingflies because between flights they are unable to stand on a surface but hang themselves from the edges of leaves or twigs using the prehensile foretarsi (Thornhill 1977; Tan and Hua 2008). *Bittacus* Latreille, 1805 is the largest and most widespread genus of Bittacidae, and comprises more than 2/3 species of the family recorded from all zoogeographical regions (Penny and Byers 1979). Owing to considerable morphological variations (Lambkin 1988; Chen et al. 2013) and complicated distribution patterns (Penny 1975; Li and Ren 2009), the evolutionary relationship within this genus remains largely unknown to date.

Chromosomes of eukaryotic organisms may carry crucial information related to the species diversification and evolution (Gokhman and Kuznetsova 2006; Noor et al. 2007; Faria and Navarro 2010). The variations of chromosome number reflect the result of complicated chromosomal rearrangements and may help reveal the evolutionary relationships of sibling species (White 1974; Lukhtanov et al. 2005; Kandul et al. 2007; Faria and Navarro 2010). The chromosomal morphology may provide substantial information related to structural rearrangements, which may contribute to the increased level of divergence among taxa (Rieseberg and Burke 2001; Navarro and Barton 2003; Butlin 2005). Such studies have been well documented in many insect groups, including aquatic bugs (Stoianova et al. 2020), psyllids (Nokkala et al. 2019), bush crickets (Kociński et al. 2018), beetles (Dutrillaux and Dutrillaux 2019), butterflies (Dincă et al. 2011), warrior wasps (Menezes et al. 2019), and ants (Pereira et al. 2018). In Bittacidae, however, the cytogenetic information is poorly documented, with only six species reported to date (Matthey 1950; Atchley and Jackson 1970; Miao and Hua 2017, 2019).

According to the limited cytogenetic data available, the chromosome number varies extensively in Bittacidae (Matthey 1950; Atchley and Jackson 1970; Miao and Hua 2017, 2019). It is 2*n* = 25 in *B. italicus* (Müller, 1766), 2*n* = 27 in *B. flavidus* Huang et Hua, 2005, 2*n* = 29 in *B. pilicornis* Westwood, 1846, 2*n* = 31 in *B. stigmaterus* Say, 1823, 2*n* = 35 in *B. planus* Cheng, 1949, and 2*n* = 41 in *Terrobittacus implicatus* (Huang et Hua in Cai et al., 2006). Each species examined has a distinctive karyotype, which represents an important diagnostic feature in Bittacidae and provides useful information on the evolutionary relationship of Mecoptera (Miao and Hua 2017, 2019).

In this paper, we present for the first time information on the karyotype and male meiosis of the hangingfly *Bittacus sinicus* Issiki, 1931, attempting to enrich our knowledge of the chromosome evolution of *Bittacus* and to contribute to the cytogenetic data for a better understanding of the evolutionary history of Bittacidae.

## Materials and methods

### Adult collecting

**Figure 1.**
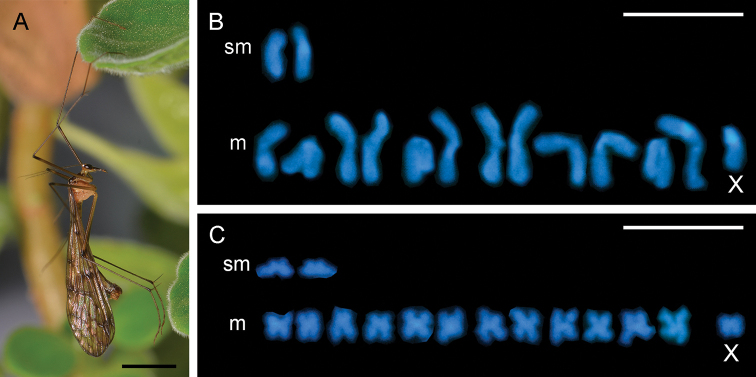
Karyotypes of *Bittacus sinicus* with DAPI staining **A** habitus of male adult **B** spermatogonial metaphase **C** meiotic anaphase I. Abbreviations: m, metacentric; sm, submetacentric; X, sex chromosome. Scale bars: 5 mm (**A**); 10 μm (**B, C**).

Adults of *B. sinicus* (Fig. 1A) were collected from Shimian County (29°03'00"N, 102°21'00"E, elev. 1800–1890 m), Sichuan Province in China from July to August in 2016 and Paomashan (30°02'36"N, 101°57'33"E, elev. 2600 m), Sichuan Province in China in late July 2018, respectively.

### Insect rearing

Live adults were reared in screen-wired cages (40 × 60 × 60 cm) containing twigs and leaves of plants and moist absorbent cotton (Miao and Hua 2019). Eggs, larvae and pupae were incubated and reared in plastic containers with humid humus. Live flies and frozen pupae of *Musca domestica* Linnaeus, 1758 (Diptera, Muscidae) were provided as food for the adults and larvae, respectively. Temperature was kept at 16 ± 2 °C for larvae, 21 ± 2 °C for pupae, and 23 ± 2 °C for adults. Relative humidity was maintained at 75 % ± 10 % (Miao and Hua 2017).

### Cytogenetic analyses

Chromosome spreads were prepared using the testes of larvae and pupae following Imai et al. (1988). The mitotic metaphase and early stages of meiosis were obtained from males of the third and fourth (last) instar larvae, and the male meiosis I/II mainly from young pupae. Totally 66 larvae (46 from Shimian County and 20 from Paomashan) and 12 pupae (nine from Shimian County and three from Paomashan) of *B. sinicus* were used for chromosome preparations.

C-banding was obtained using the same technique as in Miao and Hua (2019). The fluorochrome DAPI (4',6-diamidino-2-phenylindole) staining was performed to characterize the DAPI^+^ heterochromatin (the shiny blue regions rich in AT bases) on chromosomes, following Rebagliati et al. (2003).

Photographs were taken with a Nikon DS-Fil digital camera mounted on a Nikon Eclipse 80i microscope (Nikon, Tokyo, Japan). The fluorescence signals were observed with a UV filter (330–385 nm).

### Statistical analyses

Five spermatogonial cells with well-spread chromosomes at mitotic metaphase were used to statistically analyze the chromosomes of *B. sinicus* following the procedures of Miao and Hua (2017). The captured images were quantified using the NIS-Element D 3.22 software (Nikon, Tokyo, Japan). The chromosomal morphology was determined based on the arm ratio where chromosomes were classified as metacentric (m), submetacentric (sm), subtelocentric (st), or telocentric (t) (Levan et al. 1964). The following features of chromosomes were measured: absolute chromosome length (*AL*), long arm length (*L*), short arm length (*S*), arm ratio (*r* = *L*/*S*), centromeric index (*i* = *S* × 100/*AL*), and relative chromosome length (*RL*) of each chromosome (*RL* = *AL* × 100/∑*AL*). The evaluated data are presented as mean ± SD.

The karyotype asymmetry is represented by two components, the heterogeneous degree of chromosome lengths (interchromosomal asymmetry) and the prevalence of telo-/subtelocentric chromosomes (intrachromosomal asymmetry) (Astuti et al. 2017). Two separate parameters were assessed, i.e. Coefficient of Variation of Chromosome Length (*CV_CL_*) (Paszko 2006) and Mean Centromeric Asymmetry (*M_CA_*) (Peruzzi and Eroğlu 2013).

## Results

### Karyology

The males of *B. sinicus* possess 2*n* = 15 (Fundamental Number *FN* = 30), with the karyotype formula of 13 m + 2 sm (Fig. 1B, C).

The *AL* ranges from 7.47 ± 0.26 to 3.72 ± 0.05 μm, and the *RL* from 8.43 ± 0.29 to 4.20 ± 0.05. Autosomal bivalents decrease gradually in size, and the sex chromosome (X) is the smallest of the set. The total length of all chromosomes is 88.65 μm (Table 1).

The *M_CA_* is calculated as 12.55 and the *CV_CL_* is 19.78. The relatively low degrees of both intrachromosomal and interchromosomal asymmetries indicate that the karyotype of *B. sinicus* is almost symmetric.

**Table 1.** Morphometric analyses of the chromosomes of *Bittacus sinicus* based on five spermatogonial cells from a male larva.

Pair No.	*AL* ± SD (μm)	*RL* ± SD	*L* ± SD (μm)	*S* ± SD (μm)	(*L* – *S*)/(*L* + *S*)	*i*	*r*	Type
1	3.98 ± 0.06	4.49 ± 0.07	2.62 ± 0.05	1.36 ± 0.18	0.32	34.11	1.93	sm
	4.29 ± 0.02	4.83 ± 0.03	2.75 ± 0.03	1.53 ± 0.02	0.29	35.74	1.80	sm
2	4.97 ± 0.24	5.61 ± 0.27	2.67 ± 0.10	2.30 ± 0.10	0.07	46.27	1.16	m
	5.38 ± 0.04	6.07 ± 0.05	3.18 ± 0.22	2.20 ± 0.15	0.18	40.84	1.45	m
3	6.00 ± 0.17	6.77 ± 0.19	3.45 ± 0.05	2.55 ± 0.12	0.15	42.55	1.35	m
	6.12 ± 0.08	6.90 ± 0.09	3.35 ± 0.03	2.76 ± 0.06	0.10	45.19	1.21	m
4	6.45 ± 0.08	7.27 ± 0.09	3.48 ± 0.05	2.97 ± 0.12	0.08	46.00	1.17	m
	6.50 ± 0.21	7.33 ± 0.24	3.68 ± 0.22	2.83 ± 0.13	0.13	43.45	1.30	m
5	6.59 ± 0.15	7.44 ± 0.17	3.49 ± 0.13	3.10 ± 0.29	0.06	47.08	1.12	m
	6.60 ± 0.15	7.44 ± 0.17	3.49 ± 0.11	3.11 ± 0.20	0.06	47.16	1.12	m
6	6.92 ± 0.64	7.80 ± 0.72	3.93 ± 0.09	2.99 ± 0.12	0.14	43.18	1.32	m
	6.62 ± 0.61	7.46 ± 0.69	3.56 ± 0.26	3.05 ± 0.17	0.08	46.14	1.17	m
7	7.04 ± 0.11	7.94 ± 0.12	3.92 ± 0.09	3.12 ± 0.01	0.11	44.31	1.26	m
	7.47 ± 0.26	8.43 ± 0.29	3.97 ± 0.26	3.50 ± 0.25	0.06	46.90	1.13	m
8 (X)	3.72 ± 0.05	4.20 ± 0.05	1.98 ± 0.13	1.75 ± 0.09	0.06	46.94	1.13	m

Notes: *AL*, absolute chromosome length (actual length of chromosomes); *RL*, relative chromosome length (*RL* = *AL*/total length of the chromosome complement); SD = standard deviation; *L*, long arm length; *S*, short arm length; *i*, centromeric index (*i* = *s* × 100/*AL*); *r*, arm ratio (*r* = *L*/*S*); m, metacentric; sm, submetacentric.

### Banding patterns

**Figure 2.**
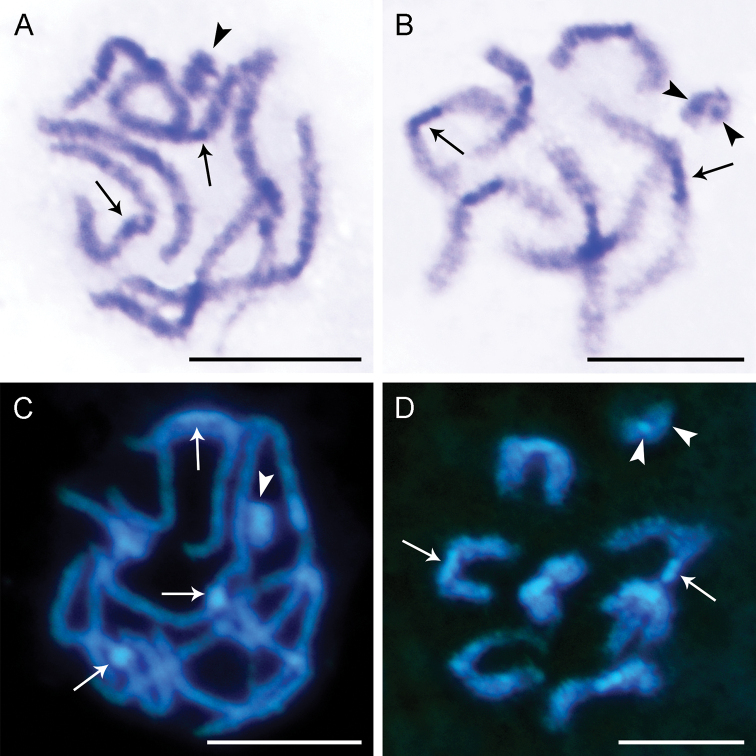
Pachytene bivalents of *Bittacus sinicus*, stained with C-banding (**A, B**) and DAPI (**C, D**) **A, C** early pachytene, showing the intermediate heterochromatin on bivalents and the heteropycnotic sex chromosome (arrowhead) **B, D** late pachytene, showing the sex chromosome with a dot-shaped heterochromatic block (arrowheads). Arrows point to the intermediate heterochromatin. Scale bars: 10 μm.

Conspicuous heterochromatin was observed on the meiotic bivalents of *B. sinicus* after C-banding and DAPI staining (Fig. 2). Both treatments reveal that the autosomal bivalents exhibit intermediate heterochromatin. The sex chromosome is heteropycnotic and totally heterochromatic at the early pachytene (Fig. 2A, C), but becomes isopycnic with two heterochromatic dots later (Fig. 2B, D).

### Chiasmate male meiosis

**Figure 3.**
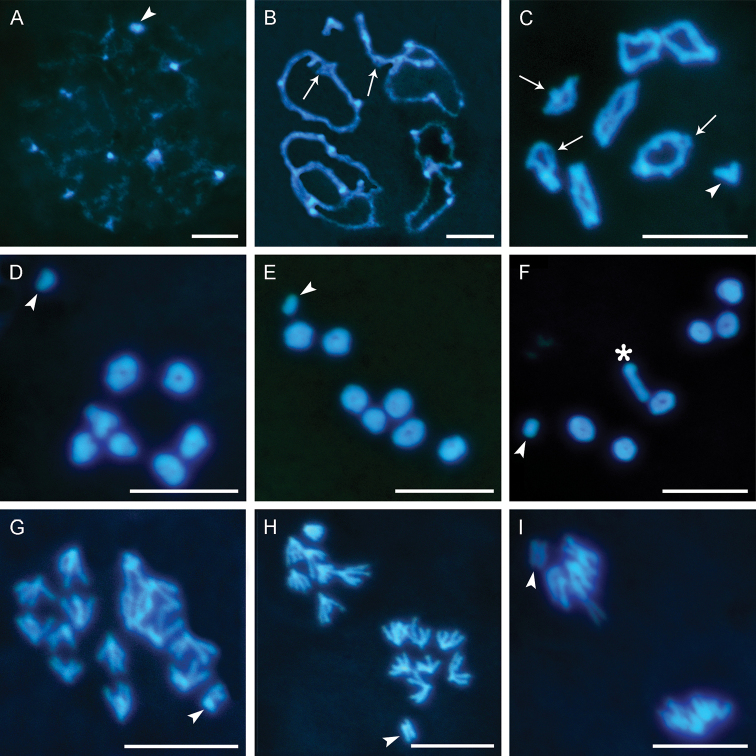
Meiosis I of *Bittacus sinicus***A** diffuse diplotene with the condensed sex chromosome and decondensed bivalents **B** diplotene, showing the bivalents are held together only at exchange points (arrows) **C** diakinesis, showing the evident chiasmata (arrows) **D** bivalents assembling at the equatorial plate in metaphase I (polar view) **E, F** metaphase I in side view, showing the ring-shaped bivalents with two chiasmata and rod-shaped bivalent with one terminal chiasma (asterisk) **G** anaphase disjunction, showing the divided bivalents and the undivided sex chromosome **H** anaphase I, showing the chromosome number of *B. sinicus* is 2*n* = 15 **I** telophase I. Arrowheads show the sex chromosome. Scale bars: 10 μm.

The synaptic attraction between the homologues terminates from the pachytene to diplotene. The early diplotene appears to be the diffuse stage, which can be interpreted as uncondensed bivalents connected by chiasmata (Fig. 3A). During this stage, the intermediate region of the bivalents is heavily stained and arranged dispersedly, while the remaining bivalents are weakly stained and are often overlooked consequently. The chromosomes move apart in repulsion and are held together only at exchange points, which appear as visible chiasmata in the diplotene stage (Fig. 3B). Metacentric bivalents exhibit two terminal chiasmata and look like large rings, whereas the submetacentric one usually contains only one terminal chiasma at the long-arm side as a long rod-shape. Chiasmata can be clearly visible after some condensation of the chromosomes at diakinesis (Fig. 3C). In *B. sinicus* the mean chiasma count per cell was 13.2 (50 cells, ranging from 13 to 14).

Bivalents assemble at the equatorial plate in metaphase I (Fig. 3D) and become oriented with their centromeres poleward (Fig. 3E). In *B. sinicus* the rod-shaped bivalent is bound by one chiasma at one arm end (asterisk in Fig. 3F), whereas the ring-shaped bivalents have both arms bound by chiasmata. The autosomal bivalents separate into dyads, whereas the X univalent moves undividedly to one pole (Fig. 3G–I), indicating that *B. sinicus* has the initial-/prereductional meiosis. Each dyad consists of two divergent chromatids associated only in the regions proximal to the centromere (Fig. 3G, H). Both submetacentric and metacentric dyads are four armed with a double V-shape in anaphase I. The dyads reach the opposite poles and fuse into an indistinguishable mass of chromatin in telophase I (Fig. 3I).

**Figure 4.**
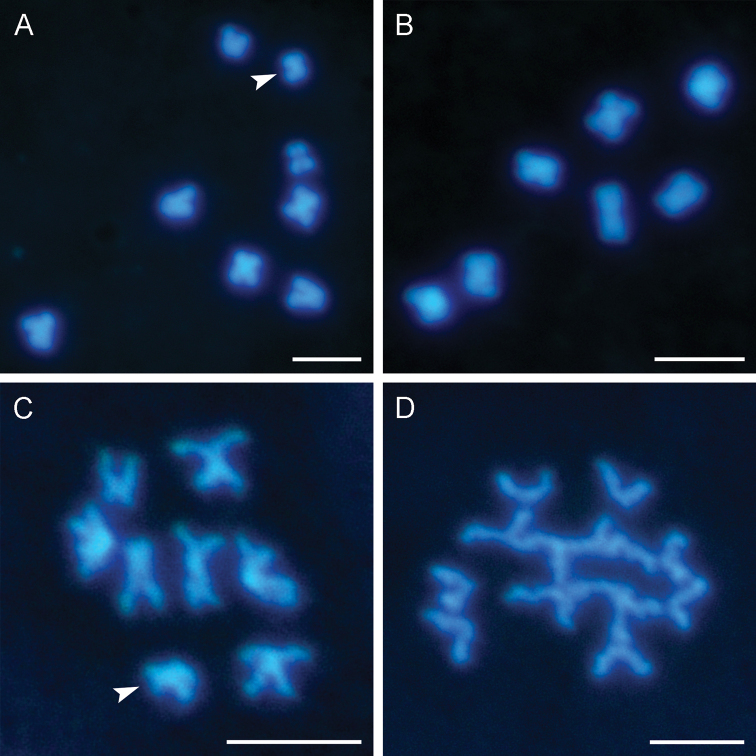
Meiosis II of *Bittacus sinicus***A, B** the secondary spermatocytes: **A** with *n* = 8 **B** with *n* = 7 **C** prometaphase II, showing the striking repulsion between the sister chromatids of each dyad chromosome **D** anaphase II, showing the separation of sister chromatids. Arrowheads show the sex chromosome. Scale bars: 5 μm.

Meiosis II takes place immediately after the first meiotic division. The movement of the X univalent toward only one pole at anaphase I leads to the formation of two classes of nuclei (Fig. 4A, B). The sister chromatids of each dyad are widely splayed, but are held together at the centromere in prometaphase II (Fig. 4C). The centromeric cohesion between the two sister chromatids is removed in anaphase II, and the sister chromatids are pulled apart by microtubules attached to the kinetochore (Fig. 4D).

### Sex chromosome system

The diploid somatic chromosome number (2*n*) is reduced to the haploid gametic chromosome number (*n*) during the first meiosis. Both the autosomes and the sex chromosome exhibit pre-reductional type of meiosis. The haploid chromosome numbers are different between the two daughter nuclei with *n* = 7 + X (Fig. 4A) and *n* = 7 (Fig. 4B), indicating an X0 sex system of the male *B. sinicus*.

## Discussion

The present study is the first attempt to investigate the karyotype and male meiosis of *B. sinicus*. As in other bittacids studied previously, *B. sinicus* has the chiasmate meiosis and the X0(♂) sex determination mechanism, which are likely the plesiomorphies in Bittacidae (Matthey 1950; Atchley and Jackson 1970; Miao and Hua 2017, 2019).

*Bittacus sinicus* has the lowest chromosome number 2*n* = 15 ever observed in Mecoptera. Previously, 2*n* = 17 chromosomes recorded for *Nannochorista dipteroides* Tillyard, 1917 (Nannochoristidae) was considered the lowest number reported for this order (Bush 1966). Despite limited chromosome data available, the chromosome number exhibits considerable variations among the families of Mecoptera, from 2*n* = 15 to 41 in Bittacidae, 2*n* = 19 to 31 in Boreidae (Cooper 1951, 1974), 2*n* = 17 to 27 in Nannochoristidae (Bush 1966), and 2*n* = 35 to 47 in Panorpidae (Naville and Beaumont 1934; Ullerich 1961; Atchley and Jackson 1970; Xu et al. 2013; Miao et al. 2017, 2019).

In Bittacidae, each species examined has a distinctive karyotype, and the two genera (*Bittacus* and *Terrobittacus* Tan et Hua, 2009) investigated are distinguishable cytogenetically. *Bittacus* has relatively low chromosome numbers and symmetric karyotypes, while *Terrobittacus* has a higher chromosome number and less symmetric karyotype (Miao and Hua 2017), suggesting that the chromosomal changes may have participated in the lineage differentiation of Bittacidae.

Interestingly, the sex chromosome is the smallest element in the karyotype of *B. sinicus*, but is larger than the majority of autosomes in other bittacids studied (Miao and Hua 2017, 2019). Therefore, we speculate that autosome-autosome fusions may contribute to the karyotype formation in *B. sinicus*. Similar rearrangements are also suggested for some recently differentiated species of the scorpionflies Panorpidae (Miao et al. 2019). A notable example is *Neopanorpa lipingensis* Cai et Hua, 2009, which has a distinct chromosome number of 2*n* = 33, not 2*n* = 41 found in most members of *Neopanorpa* van der Weele, 1909, indicating that fusion events occurred at least eight times among the autosomes.

The C-banding pattern of *B. sinicus* is represented by intermediate blocks on pachytene bivalents and is definitely different from the heterochromatic segment at one bivalent terminal in other bittacids (Atchley and Jackson 1970; Miao and Hua 2017, 2019), implying that inversions may participate in the changes of chromosome morphology.

Conspicuous bands are detectable on pachytene bivalents using the DAPI staining. In general, the terminal DAPI^+^ (AT-rich) heterochromatin at one side of a bivalent is the most frequent pattern, which has been observed in the majority of Panorpidae and Bittacidae investigated (Miao and Hua 2017, 2019; Miao et al. 2019). In *B. sinicus*, however, the DAPI^+^ bands are present in the intermediate regions of all bivalents (Fig. 2C, D). Bivalents with intermediate DAPI^+^ heterochromatin were also found in the species of *Neopanorpa* and were considered as important evidence for the evolutionary reduction of chromosome number in Panorpidae (Miao et al. 2019).

Two alternative hypotheses (fission and fusion) can explain the karyotype formation in the genus *Bittacus*. The fission hypothesis assumes that the cytogenetic features of *B. sinicus* are primitive with a low chromosome number, relatively large autosomes and reduced heterochromatin. The karyotype changes of *Bittacus* (Miao and Hua 2017, 2019) are similar to those of ants and wasps, in which the centric fissions tend to increase the chromosome number and accumulate chromatin (mainly heterochromatin) (Imai et al. 1986, 1994, 2001).

Alternatively, the fusion hypothesis may also explain the karyotype variations found in *Bittacus*. The karyotype of *B. sinicus* is considered the derived condition and is shaped by Robertsonian translocations of acrocentric chromosomes and/or reciprocal translocations between meta-/submetacentric and acrocentric ones, which are generated by pericentric inversions. During the translocation events, small centromeric chromosomes (in addition to the final fused chromosomes) may be produced and lost within a few cell cycles. Such scenarios may explain the elimination of centromeres and heterochromatin toward the *B. sinicus* karyotype, and has been suggested for many monocentric organisms, such as the plant *Arabidopsis thaliana* (Linnaeus, 1758) (Lysak et al. 2006), the flatworm *Aspidogaster limacoides* Diesing, 1834 (Bombarová et al. 2015), the pangolin *Manis javanica* (Desmarest, 1822) (Nie et al. 2009), the mouse *Akodon* Meyen, 1833 (Ventura et al. 2009), the grasshopper *Ronderosia* Cigliano, 1997 (Orthoptera, Acrididae) (Castillo et al. 2019), the beetle *Dichotomius* Hope, 1838 (Coleoptera, Scarabaeidae) (Cabral-de-Mello et al. 2011), and the ants Myrmicinae (Cardoso et al. 2014). Based on the phylogeny of the Chinese Bittacidae (YM, unpublished data), we speculate that the cytogenetic features observed in *B. sinicus* may be derived conditions, including the low number of chromosomes, relatively large sizes of autosomes and the intermediate distribution of heterochromatin.

Chromosome rearrangements are proposed as an important driving force of diversification since they lead to speciation via formation of reproductive incompatibility or recombination suppression (Navarro and Barton 2003; Ayala and Coluzzi 2005; Butlin 2005; Kandul et al. 2007; Brown and O’Neill 2010; Kirkpatrick 2010; Mills and Cook 2014). According to the models of chromosomal speciation, there is an increasing level of divergence near rearrangement breakpoints, which tend to accumulate alleles involved in the reproductive isolation (Coghlan et al. 2005; Faria and Navarro 2010). In *Bittacus*, the cytogenetic data available indicate that the chromosomal evolution involves progressive changes in chromosome number and karyotype structure. However, it remains unclear whether these chromosomal rearrangements are an integral component and driving force of the speciation process or they are established later, after speciation is completed. Further investigations of additional species, combined with molecular phylogeny and fluorescent in situ hybridization (telomere and 18S rDNA probes), are needed to shed more light on this issue.
